# Supplementary material for: The microRNA cluster miR-30b/-30d prevents tumor cell switch from an epithelial to a mesenchymal-like phenotype in GBC
Source: Mol Ther Methods Clin Dev. 2020 Dec 3;20:716–25. doi: 10.1016/j.omtm.2020.11.019 (PMC7937539; doi:10.1016/j.omtm.2020.11.019)
Supplement: Document S1. Figure S1 [file mmc1.pdf]

OMTM, Volume *20*

Supplemental information

The microRNA cluster miR-30b/-30d prevents tumor cell switch from an epithelial to a mesenchymal-like phenotype in GBC

Kang Cui and Xinyan Bian

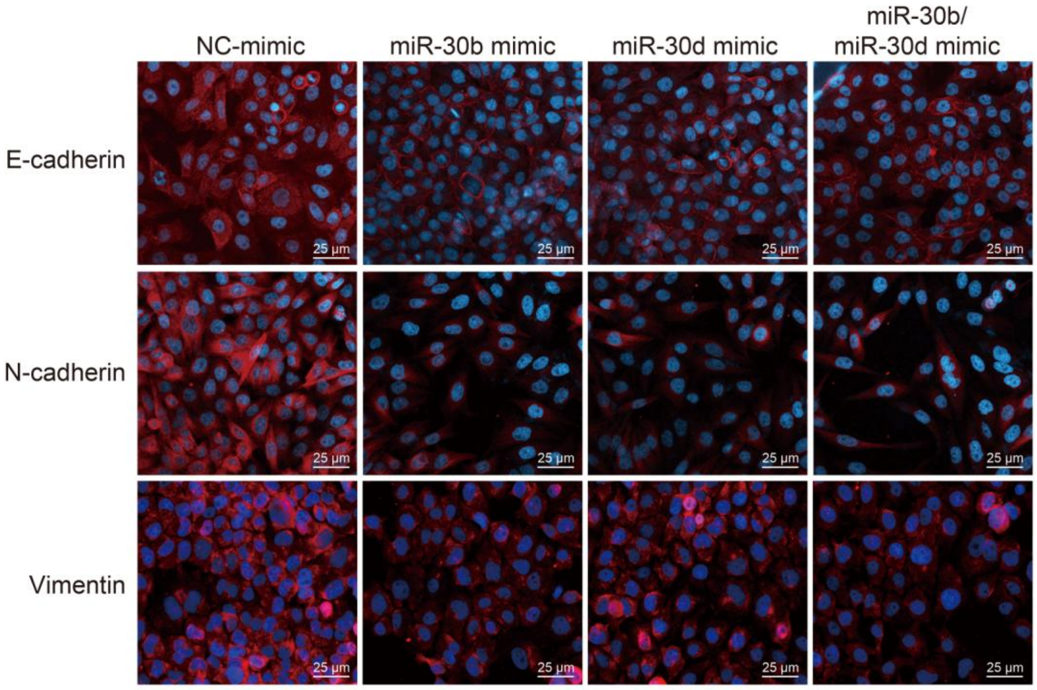

Supplementary Figure 1 Representative view of GBC-SD cells probed with antibodies against E-cadherin, N-cadherin, and vimentin and visualized by immunofluorescence (×400).
