# Supplementary material for: The microRNA cluster miR-30b/-30d prevents tumor cell switch from an epithelial to a mesenchymal-like phenotype in GBC
Source: Mol Ther Methods Clin Dev. 2020 Dec 3;20:716–25. doi: 10.1016/j.omtm.2020.11.019 (PMC7937539; doi:10.1016/j.omtm.2020.11.019)
Supplement: Document S2. Article plus supplemental information [file mmc2.pdf]

Molecular Therapy
Methods & Clinical Development
Original Article

The microRNA cluster miR-30b/-30d prevents tumor cell switch from an epithelial to a mesenchymal-like phenotype in GBC

Kang Cui[1] and Xinyan Bian[2]

[1]Clinical Laboratory, Linyi People's Hospital, Linyi 276003, P.R. China; [2]Anorectal Branch, Linyi People's Hospital, Linyi 276003, P.R. China

As a malignancy of the gastrointestinal tract, gallbladder cancer (GBC) continues to exhibit notable rates of mortality. The current study aimed at investigating the effects associated with miR-30b and miR-30d (miR-30b/-30d) patterns in tumor cells undergoing epithelial-to-mesenchymal transition (EMT) in GBC. It identified that miR-30b and miR-30d, composed as a miRNA cluster, exhibited lower levels in the cancerous tissues from 50 patients with GBC relative to the gallbladder tissues from 35 patients with chronic cholecystitis. As expected, elevated expression of miR-30b/-30d was found to inhibit the EMT process, as evidenced by enhanced E-cadherin and reduced N-cadherin and vimentin in human GBC cells treated with miR-30b mimic, miR-30d mimic, and miR-30b/-30d mimic. Semaphorin-6B (SEMA6B) was identified as a target gene of miR-30b/-30d. Silencing of SEMA6B by its specific small interfering RNA (siRNA) mimicked the effect of miR-30b/-30d upregulation on the GBC cell EMT. Consistently, SEMA6B overexpression promoted this phenotypic switch even in the presence of miR-30b/-30d mimic. The tumorigenicity assay data obtained from nude mice also further supported the notion that miR-30b/-30d inhibited EMT of GBC cells. Thus, based on the key findings of the current study, we concluded that the miR-30b/-30d cluster may provide a potential avenue for targeting mesenchymal-like, invasive tumor cells in GBC.

INTRODUCTION

Gallbladder cancer (GBC) represents a well-documented malignancy characterized by its aggressive nature, notable regional lymph node metastasis, and an early stage distant site metastasis, with previous data suggesting approximately 219,420 newly diagnosed cases and 165,087 deaths reported in 2018 in 185 countries.[1,2] GBC is a malignancy of the biliary tract that has been reported to occur at higher rates in developing countries, with the current available treatment modalities often proving to be insufficient, particularly in patients diagnosed at a later stage of disease.[3] Thus, it is imperative that molecular factors associated with GBC continue to be explored with the objective of identifying more effective and beneficial therapeutic targets for GBC.

A recent increase in research attention has occurred due to the proposed roles of microRNAs (miRNAs or miRs) in GBC owing to their aberrant expression profile in GBC tissues and regulatory roles in cell malignant behaviors.[4,5] miRNAs, with their lengths ranging from 18 to 24 nt, have been highlighted as either tumor suppressors or oncogenes based on their binding to target messenger RNAs (mRNAs) and regulation of cancer progression, drug resistance, metastasis, or relapse.[6] For instance, miR-125b has been reported to function as an independent biomarker for oncologic outcomes of patients with GBC by exerting tumor-suppressive effects.[7] Evidence has highlighted the role of miR-33a in GBC progression by means of GBC epithelial-mesenchymal transition (EMT) regulation and cell proliferation, migration, and invasion abilities both *in vitro* and *in vivo*.[8] As a distinctive feature of carcinogenesis, EMT has been identified as an evolutionarily conserved process with significant capacity to mediate carcinogenesis and metastasis, highlighting its potential as a therapeutic candidate against cancer development.[9] Based on microarray-based expression analysis obtained prior to our investigation, miR-30b/-30d was revealed to form a cluster with significantly low expression in GBC samples, which represents a significant finding, peaking our research interests into further investigation into the functional role and the underpinning mechanism by which miR-30b/-30d contributes to GBC. Evidence has been presented emphasizing the role of miR-30b in methylglyoxal-induced EMT process in peritoneal mesothelial cells of rats.[10] Additionally, studies have indicated the ability of miR-30d to inhibit the initiation of EMT by transforming growth factor β1 (TGF-β1) in ovarian cancer cells.[11] Importantly, the synergistic effects of miR-30b/-30d have been validated in metastatic potential and cellular invasion of human melanoma.[12] Hence, we inferred that miR-30b/-30d may also exert synergistic effects in the regulation of EMT in GBC. Moreover, the presence of binding sites between miR-30b/-30d and human semaphorin-6B (SEMA6B) was predicted through *in silico* analysis (http://www.mirdb.org/). As a member of the semaphoring axon-guidance family, SEMA6B has been implicated in the tumorigenesis of breast cancer as well as involvement in human umbilical vein endothelial cells.[13,14] On the basis of published data, we conducted an investigation into the less

Received 26 July 2020; accepted 24 November 2020;
https://doi.org/10.1016/j.omtm.2020.11.019.

Correspondence: Xinyan Bian, Anorectal Branch, Linyi People's Hospital, No. 27, East Section of Jiefang Road, Linyi 276003, Shandong Province, P.R. China.
E-mail: lybianxinyan@163.com

 © 2021 The Authors.
This is an open access article under the CC BY license (http://creativecommons.org/licenses/by/4.0/).

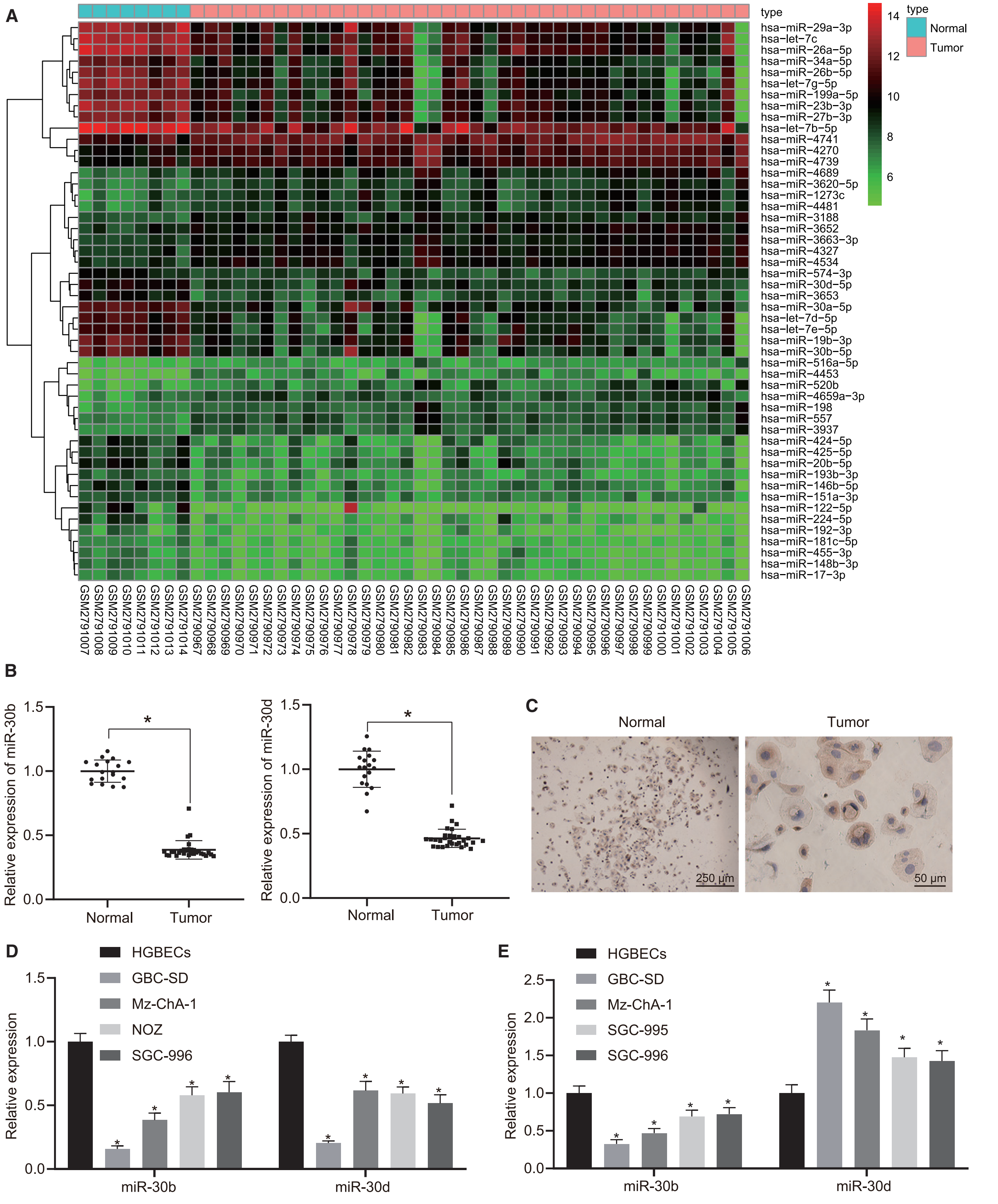

(legend on next page)

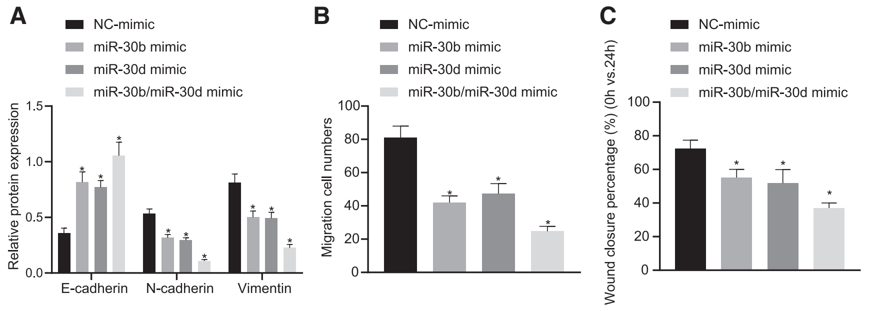

Figure 2. The miR-30b/-30d miRNA cluster represses the EMT process in GBC cells

The miR-30b mimic, miR-30d mimic, and miR-30b/-30d mimic were introduced into GBC-SD cells to elevate the expression of miR-30b, miR-30d, and miR-30b/-30d. (A) Quantification of E-cadherin, N-cadherin, and vimentin proteins in GBC-SD cells. (B) Statistics of GBC-SD cells invading from Matrigel-coated upper Transwell chambers into lower ones. (C) Statistics of GBC-SD cell migration. *p < 0.05 compared with GBC-SD cells treated with miRNA-mimic NC by independent sample t test.

studied role of SEMA6B in GBC through its interplay with miR-30b/-30d by regulating EMT.

RESULTS

The miR-30b/-30d miRNA cluster was downregulated in GBC

We initially set out to analyze the differentially expressed miRNAs between 40 GBC and 8 normal gallbladder tissue samples in the GEO: GSE104165 dataset deposited in the Gene Expression Omnibus (GEO) database, with 358 differentially expressed miRNAs identified (Figure 1A). Using the public database miRBase (http://www.mirbase.org/cgi-bin/mirna_entry.pl?acc=MI0000255), miR-30b and miR-30d, belonging to a miRNA cluster, were both shown to be downregulated in GBC tissues. In order to prove the hypothesis that downregulation of the miR-30b/-30d miRNA cluster is associated with the development of GBC, we quantified the expression of miR-30b/-30d in 30 cancerous tissues and 18 normal gallbladder tissues by quantitative reverse transcription polymerase chain reaction (qRT-PCR). As expected, the results obtained illustrated that miR-30b and miR-30d both exhibited lower expression levels in GBC tissues than in normal gallbladder tissues ($p < 0.05$, Figure 1B). The epithelial nature of the human gallbladder epithelial cells (HGBECs) was confirmed by immunohistochemical staining for CK19 (Figure 1C). HGBECs presented a polygonal and flat shape and grew to confluent monolayers.

In parallel, the expression of miR-30b and miR-30d was further evaluated in cultured GBC cells (GBC-SD, Mz-ChA-1, NOZ, and SGC-996) and HGBECs. As we expected, miR-30b and miR-30d both exhibited lower expression levels in all GBC cells when compared to that in the HGBECs, with the GBC-SD cells exhibiting the lowest expression ($p < 0.05$, Figure 1D). Furthermore, E-cadherin was found to exhibit low levels of expression while vimentin was highly expressed in GBC cells relative to HGBECs, with GBC-SD cells displaying the most significant variation ($p < 0.05$, Figure 1E). Thus, GBC-SD cells were selected for subsequent investigation. The aforementioned findings highlighted the poor expression of the miR-30b/-30d miRNA cluster in GBC.

The miR-30b/-30d miRNA cluster inhibited the EMT in GBC cells

We subsequently evaluated the effects associated with the miR-30b/-30d miRNA cluster on GBC, with a specific focus on the phenotypic switch of the GBC cells from an epithelial to a mesenchymal phenotype. With this in mind, we introduced the miR-30b mimic, miR-30d mimic, and miR-30b/-30d mimic into GBC-SD cells and examined the expression of EMT-specific markers, E-cadherin, N-cadherin, and vimentin. The immunofluorescence and immunoblot analysis results (Figures 2A, 2B, and S1) illustrated that miR-30b mimic, miR-30d mimic, and miR-30b/-30d mimic all led to increased endogenous expression of E-cadherin while reducing N-cadherin and vimentin, suggesting that the miR-30b/-30d miRNA cluster inhibited the EMT in GBC cells. Accordingly, the scratch test and Transwell invasion assays (Figures 2C and 2D) demonstrated that the miR-30b mimic, miR-30d mimic, and miR-30b/-30d mimic all weakened GBC-SD cell invasion and migration. However, no significant difference was detected regarding the expression of EMT-specific markers and GBC-SD cell invasion and migration in cells treated with miR-30b mimic, miR-30d mimic, and miR-30b/-30d mimic. Altogether, the results obtained suggested that the miR-30b/-30d miRNA cluster could suppress the EMT and invasion and migration of GBC cells.

SEMA6B was a key underlying target of the miR-30b/-30d miRNA cluster

We subsequently set out to elucidate the mechanism by which the miR-30b/-30d miRNA cluster inhibited EMT in GBC cells. The possible target genes of miR-30b and miR-30d were identified from four public databases, i.e., DIANA, miRDB, mirDIP, and TargetScan. Figure 3A depicts the relevant target genes meeting >80 scores (the miRDB database) as well as the top 400 scores (other three databases). Evaluation of the differentially expressed mRNAs between three pairs of human GBC and matched paracancerous tissues in the

Figure 1. The miR-30b/-30d miRNA cluster is downregulated in GBC

(A) Heatmap presenting the top 50 miRNAs with the most significant expression difference between GBC (n = 40) and normal gallbladder (n = 8) tissue samples in the GEO: GSE104165 dataset. Color scale indicates the degree of upregulation (red) or downregulation (green). (B) Expression of miR-30b/-30d in cancerous tissues (n = 30) and normal gallbladder tissues (n = 18), normalized to that of U6. (C) Epithelial nature of HGBECs (left map: original magnification, ×40; right map: original magnification, ×200). (D) Expression of miR-30b/-30d in cultured GBC cells (GBC-SD, Mz-ChA-1, NOZ, and SGC-996) and HGBECs. (E) The expression of E-cadherin and vimentin in cultured GBC cells (GBC-SD, Mz-ChA-1, NOZ, and SGC-996) and HGBECs. *p < 0.05 compared with the corresponding control (independent sample t test for statistical comparisons between two groups and one-way ANOVA followed by Tukey's test among multiple groups).

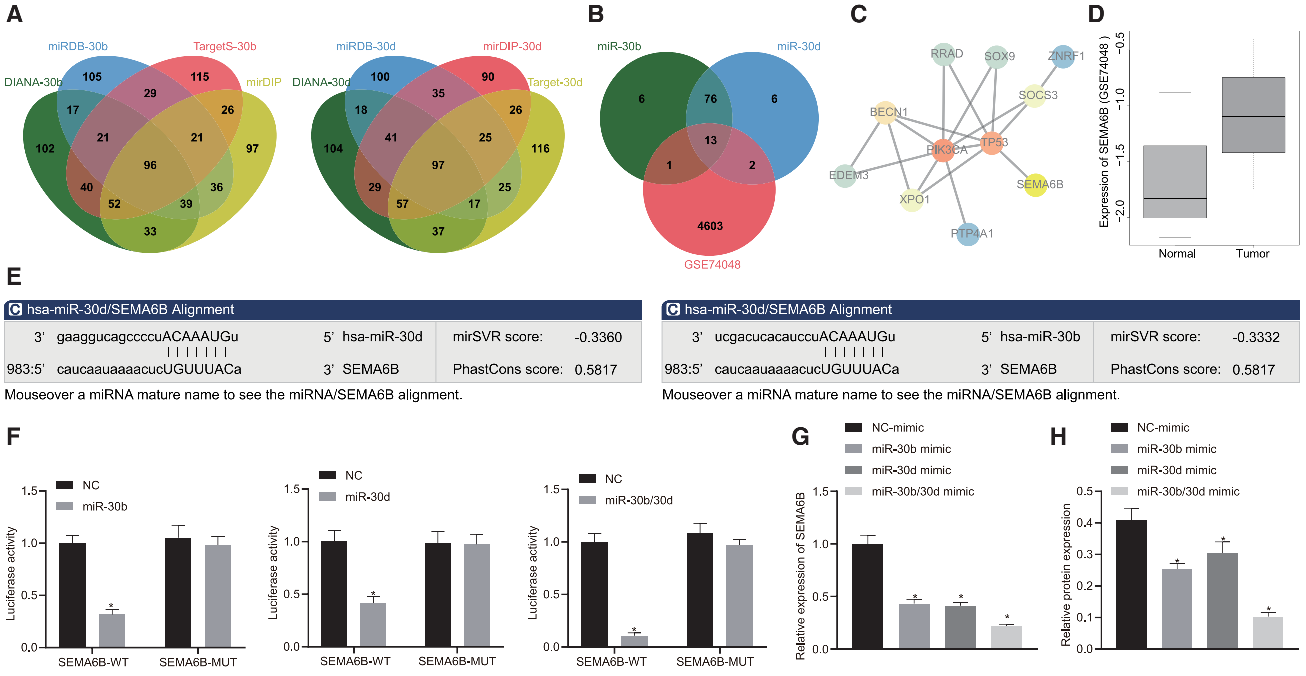

Figure 3. The miR-30b/-30d miRNA cluster targets and negatively regulates the expression of SEMA6B

(A) Target genes of miR-30b and miR-30d obtained from four public databases, including DIANA (the top 400 scores, http://diana.imis.athena-innovation.gr/DianaTools/index.php?r=microT_CDS/index); miRDB (>80 scores, http://mirdb.org/miRDB/index.html); mirDIP (the top 400 scores, http://ophid.utoronto.ca/mirDIP/index.jsp#r); and TargetScan (the top 400 scores, http://www.targetscan.org/vert_71/). (B) Overlapping target genes of miR-30b/-30d from the GEO: GSE74048 dataset and four public databases by Venn diagrams (http://jvenn.toulouse.inra.fr/app/example.html). (C) Interactions between 10 recognized GBC-associated genes (the MalaCards database, https://www.malacards.org/) and 13 miR-30b/-30d-binding downstream genes predicted using the STRING database (https://string-db.org/). (D) Differential analysis of SEMA6B expression between human GBC (n = 3) and matched paracancerous (n = 3) tissues in the GEO: GSE74048 dataset. (E) The predicted seed sequence of miR-30b/-30d and SEMA6B in the miRNA target detection program (http://www.mirdb.org/). (F) Luciferase activity of SEMA6B-WT and SEMA6B-MUT in HEK293T cells transfected with miR-30b mimic, miR-30d mimic, or miR-30b/-30d mimic. (G) mRNA expression of SEMA6B in GBC-SD cells transfected with miR-30b mimic, miR-30d mimic, or miR-30b/-30d mimic. (H) Protein expression of SEMA6B in GBC-SD cells transfected with miR-30b mimic, miR-30d mimic, or miR-30b/-30d mimic. *$p < 0.05$ compared with the corresponding control (independent sample t test for statistical comparisons between two groups and one-way ANOVA followed by Tukey's test among multiple groups).

GEO: GSE74048 dataset was then performed. The upregulated mRNAs in the GEO: GSE74048 dataset were overlapped with the miR-30b/-30d-binding downstream genes predicted by the Venn diagrams, which revealed 13 miR-30b/-30d-binding downstream genes related to GBC (Figure 3B). Ten GBC-associated genes were identified from the MalaCards database and established gene-gene models to discover interactions between multiple genes in the STRING database. Figure 3C illustrates an interaction between the tumor protein p53 (TP53) gene (at the center in the regulatory network) and SEMA6B. SEMA6B was an upregulated mRNA in the GEO: GSE74048 dataset (Figure 3D). Hence, we asserted the hypothesis that the miR-30b/-30d miRNA cluster may target and negatively regulate the expression of SEMA6B. To address it, we first used a computer-based miRNA target detection program to obtain the predicted seed sequence of miR-30b/-30d and SEMA6B (Figure 3E). Luciferase activity at the promoter of the reporter gene inserted with the seed sequence in the 3′ untranslated region (UTR) of SEMA6B instead of the relative mutant reporter gene was reduced in the presence of miR-30b mimic, miR-30d mimic, or miR-30b/-30d mimic (Figure 3F). The expression of SEMA6B was subsequently analyzed in cultured GBC-SD cells at the mRNA level (qRT-PCR) and protein level (immunoblot analysis) in the presence of miR-30b mimic, miR-30d mimic, or miR-30b/-30d mimic. Our data revealed that elevated miR-30b/-30d triggered a decrease in the expression of SEMA6B (Figures 3G and 3H). Altogether, the miR-30b/-30d miRNA cluster could target and negatively regulate the expression of SEMA6B.

Silencing SEMA6B inhibited the EMT in GBC cells

Next, to evaluate SEMA6B in GBC, we analyzed the expression pattern of SEMA6B in GBC tissues relative to normal gallbladder tissues and GBC-SD cells relative to HGBECs by qRT-PCR and immunoblot analysis. The results obtained indicated that the mRNA and protein expression levels of SEMA6B were both upregulated in GBC tissues and GBC-SD cells relative to normal gallbladder tissues and HGBECs, respectively (Figures 4A–4D). In the subsequent experiments, we specifically blunted the expression of SEMA6B in GBC cells by delivering small interfering RNA (siRNA) targeting SEMA6B into GBC-SD cells and then examined the EMT, cell invasion, and migration. The immunoblot analysis revealed enhanced E-cadherin protein expression, along with diminished N-cadherin and vimentin

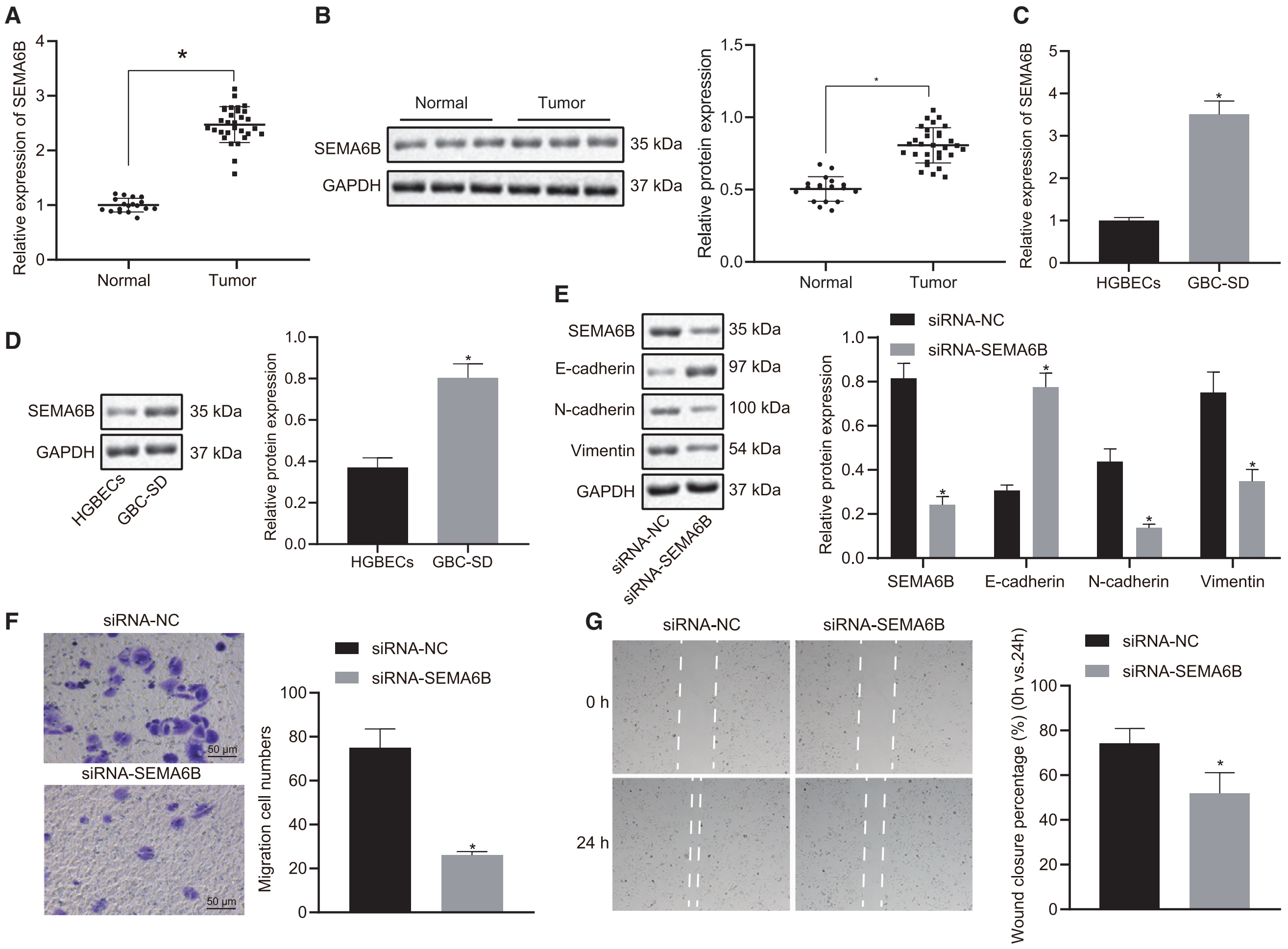

Figure 4. Silencing of SEMA6B inhibits the EMT process in GBC cells

(A) mRNA expression of SEMA6B in GBC tissues relative to normal gallbladder tissues. (B) Protein expression of SEMA6B in GBC tissues relative to normal gallbladder tissues. (C) mRNA expression of SEMA6B in GBC cells relative to HGBECs. (D) Protein expression of SEMA6B in GBC cells relative to HGBECs. In the following experiments, siRNA targeting SEMA6B and scramble siRNA were delivered into GBC-SD cells to specifically blunt the expression of SEMA6B. (E) Immunoblots and quantification of E-cadherin, N-cadherin, and vimentin proteins in GBC-SD cells. (F) Representative view and statistics of GBC-SD cells invading from Matrigel-coated upper Transwell chambers into lower ones. (G) Representative view and statistics of GBC-SD cell migration. *$p < 0.05$ compared with GBC-SD cells treated with scramble siRNA by an independent sample t test.

protein expression in GBC-SD cells with SEMA6B knockdown (Figure 4E). Scratch test and Transwell invasion assays (Figures 4F and 4G) demonstrated that siRNA-mediated knockdown of SEMA6B weakened GBC-SD cell invasion and migration. The aforementioned results suggested that upregulation of SEMA6B might be associated with the development of GBC, and silencing of SEMA6B mimicked the effect of miR-30b/-30d elevation on GBC.

The miR-30b/-30d cluster prevented EMT by repressing SEMA6B expression

Owing to the reciprocal regulation that exists between the miR-30b/-30d cluster and SEMA6B, rescue assays were conducted to verify the final hypothesis that the miR-30b/-30d cluster prevents the EMT process by downregulating SEMA6B expression. GBC-SD cells treated with miR-30b/-30d mimic were transfected with an expression vector containing the SEMA6B gene to restore the expression of SEMA6B (Figures 5A and 5B). Likewise, the immunoblot analysis results demonstrated that restoration of SEMA6B expression triggered a decline in the protein expression of E-cadherin, as well as an enhancement of N-cadherin and vimentin protein expression in GBC-SD cells treated with miR-30b/-30d mimic (Figure 5C). Accordingly, a scratch test and Transwell invasion assays (Figures 5D and 5E) demonstrated that restoration of SEMA6B expression brought about enhanced invasion and migration abilities of GBC-SD cells treated with miR-30b/-30d mimic. The aforementioned findings provided evidence supporting the hypothesis we initially proposed that the miR-30b/-30d cluster prevented the EMT process by downregulating SEMA6B expression.

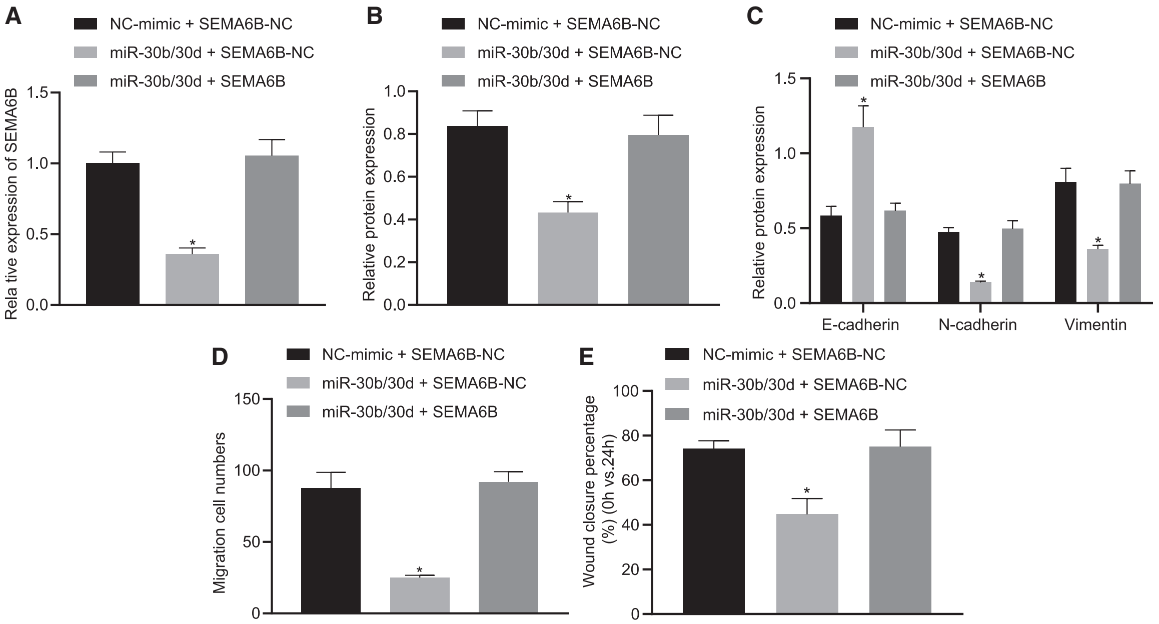

Figure 5. The miR-30b/-30d cluster prevents the EMT process by repressing SEMA6B expression

GBC-SD cells treated with miR-30b/-30d mimic (miR-mimic NC as negative control) were transfected with an expression vector containing the SEMA6B gene (empty vector as negative control). (A) mRNA expression of SEMA6B in GBC-SD cells. (B) Protein expression of SEMA6B in GBC-SD cells. (C) Quantification of E-cadherin, N-cadherin, and vimentin proteins in GBC-SD cells. (D) Statistics of GBC-SD cells invading from Matrigel-coated upper Transwell chambers into lower ones. (E) Statistics of GBC-SD cell migration. *p < 0.05 compared with the corresponding control by one-way ANOVA followed by Tukey's test.

The miR-30b/-30d cluster retarded tumorigenesis and EMT of human GBC cells *in vivo* by downregulating SEMA6B

Finally, we established subcutaneous xenotransplanted tumor models of untreated GBC-SD cells, miR-30b/-30d mimic-treated GBC-SD cells, and GBC-SD cells treated with miR-30b/-30d mimic in addition to an expression vector containing the SEMA6B gene. As shown in Figures 6A and 6B, the results obtained highlighted that elevated miR-30b/-30d inhibited the growth of the subcutaneous xenotransplanted tumors of human GBC-SD cells. However, overexpression of SEMA6B was observed to restore the tumorigenesis of miR-30b/-30d mimic-treated GBC-SD cells in nude mice. We subsequently set out to ascertain the protein expression of SEMA6B, E-cadherin, N-cadherin, and vimentin in tissues derived from subcutaneous xenotransplanted tumors via immunoblot (Figure 6C) and immunohistochemistry (Figure 6D) analyses. Both sets of results suggested that elevated miR-30b/-30d enhanced the protein expression of E-cadherin, while reducing that of SEMA6B, N-cadherin, and vimentin in subcutaneous xenotransplanted tumor tissues, which was abrogated by the overexpression of SEMA6B. Thus, we arrived at the conclusion that the miR-30b/-30d cluster inhibited tumorigenesis and EMT of human GBC cells *in vivo* by downregulating SEMA6B.

DISCUSSION

Accumulating evidence continues to emphasize the importance of various miRNAs in GBC with studies highlighting their relationship with the malignant behaviors displayed by cancer cells and correlation to poor oncologic outcomes of patients with GBC.[15–17] The present study set out to elucidate the role of the functional miRNA cluster in connection with miR-30b/-30d in GBC via SEMA6B targeting. Collectively, the experimental data demonstrated that the forced expression of miR-30b/-30d possessed the capacity to impede the EMT of GBC cells *in vitro* and tumorigenesis *in vivo* through downregulation of SEMA6B.

A fundamental finding of our study indicated that miR-30b/-30d was poorly expressed in GBC tissues and cells. Low expression of miR-30b has been highlighted as an indicator of undesirable overall survival of patients with esophageal cancer while esophageal cancer cell growth, migration, and invasion are significantly suppressed in the setting of miR-30b overexpression.[18] The dysregulation of miR-30b has also been identified in non-small cell lung cancer (NSCLC) and colorectal cancer while the restoration of miR-30b expression ameliorates tumorigenic characteristics.[19,20] Likewise, low miR-30d-5p expression in GBC tissues has been shown to be highly suggestive of poor prognosis of patients with GBC in contribution to aggressive progression of GBC.[21] Additionally, the downregulation of miR-30b/-30d has been detected in ovarian cancer cells with the delivery of miR-30d mimic shown to upregulate the expression of E-cadherin while downregulating N-cadherin expression, impeding the EMT process induced by TGF-β1.[11] A corresponding investigation was followed in our study, with the results demonstrating that restoration of miR-30b by miR-30b mimic, restoration of miR-30d by miR-30d mimic, or restoration of miR-30b/-30d by miR-30b/-30d mimic could all reverse EMT phenotypes in GBC, including the expression pattern

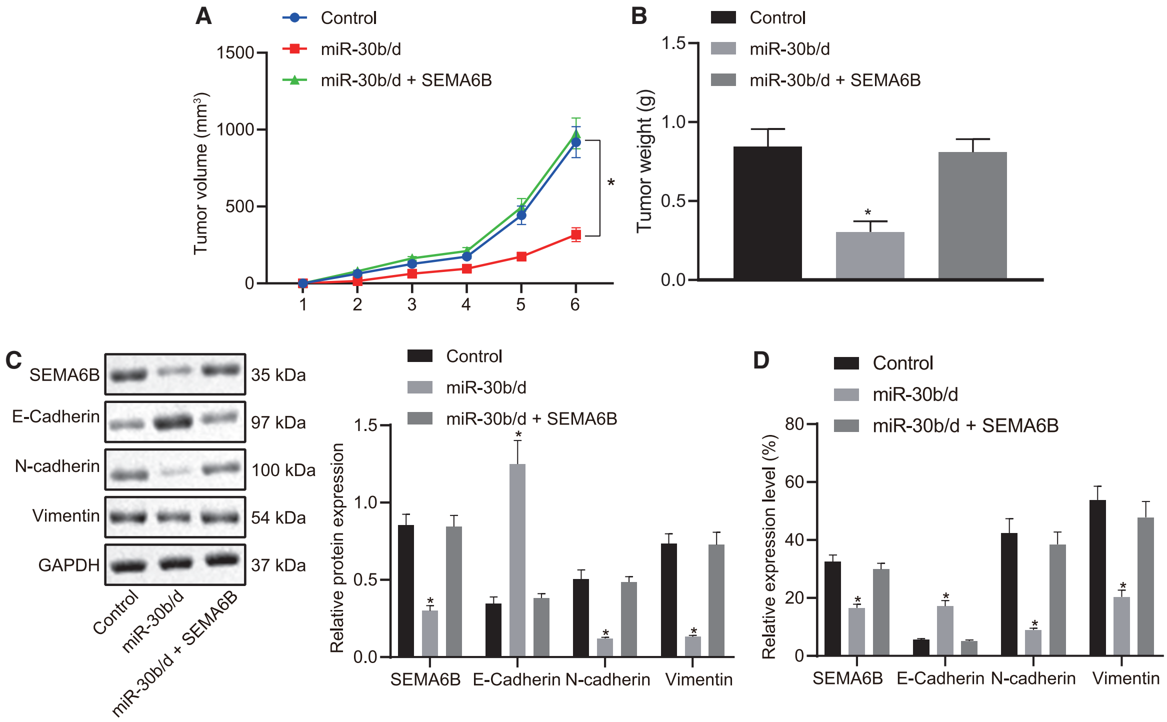

Figure 6. miR-30b/-30d cluster inhibits tumorigenesis and EMT of human GBC cells *in vivo* by downregulating SEMA6B

(A) Volume of mouse xenotransplanted tumors of GBC-SD cells at indicated time points; a repeated-measures ANOVA with a Bonferroni test was used for statistical comparison. (B) Weight of mouse xenotransplanted tumors of GBC-SD cells. (C) Immunoblots and quantification of SEMA6B, E-cadherin, N-cadherin, and vimentin proteins in mouse tumor tissues. (D) Immunohistochemical staining for SEMA6B, E-cadherin, N-cadherin, and vimentin proteins in mouse tumor tissues and extent of staining. *$p < 0.05$ compared with the corresponding control by one-way ANOVA followed by Tukey's test.

of molecular markers (E-cadherin, N-cadherin, and vimentin) and migratory and invasive capabilities in GBC cells. Largely consistent with our findings, the overexpression of miR-30b has been demonstrated to result in a decreased level of mesenchymal marker N-cadherin and increased level of the epithelial marker E-cadherin, impairing the invasive and migration capacities as well as the tumorigenic potential of pancreatic cancer stem cells.[22] Notably, a significant relevance of miR-30b/miR-340 with the EMT process in GBC has been unveiled that malignant phenotypes of GBC cells can be suppressed in the setting of miR-30b/miR-340 overexpression accompanied by a decrease in expression of vimentin and promoted expression of E-cadherin.[23] High levels of miR-30d/miR-30d-5p expression have been confirmed to act as a tumor suppressor by inhibiting cancer cell motility in esophageal squamous cell carcinoma and NSCLC.[24,25] Notably, a miRNA-miRNA pair has been recognized as a functional synergistic network identified in a wide array of malignancies.[26–28] This discovery enhanced our understanding of the complex regulation of miRNA-miRNA interaction, highlighting its potential as a new therapeutic target for GBC.

Furthermore, our data suggested that SEMA6B, as a target of miR-30b/-30d, was highly expressed in GBC tissues and cells, while the re-introduction of SEMA6B was found to counteract the inhibitory effect of miR-30b/-30d on GBC cell migration, invasion, as well as the EMT process. Upregulation of SEMA6B has been identified in gastric cancer tissues and correlated with the process of tumor differentiation and distant metastasis, while the processes of tumor cell migration, invasion, and adhesion can be restrained by silencing of SEMA6B.[29] In addition, the tumor formation ability of U87MG glioblastoma cells can be crippled by silencing of SEMA6B.[14] Moreover, miRNA biogenesis along with the underlying regulatory mechanisms in the initiation and development of biliary tract cancers has been linked with altered expression of their target genes.[30] Similarly, the ectopic expression of miR-30b/-30d has been demonstrated to enhance the metastatic properties of melanoma cells by binding to GALNT7, while restoration of the expression of GALNT7 has been reported to reverse the results triggered by the upregulation of miR-30d,[12] supporting the notion that SEMA6B contributes to the regulatory functions of miR-30b/-30d in the progression of GBC.

In conclusion, the key findings of the present study support the hypothesis that overexpression of miR-30b/-30d exerted synergistic effects on GBC progression by inhibiting GBC cell migration, invasion, and the EMT process *in vitro* as well as weakening tumorigenesis of GBC cells *in vivo* through target inhibition of

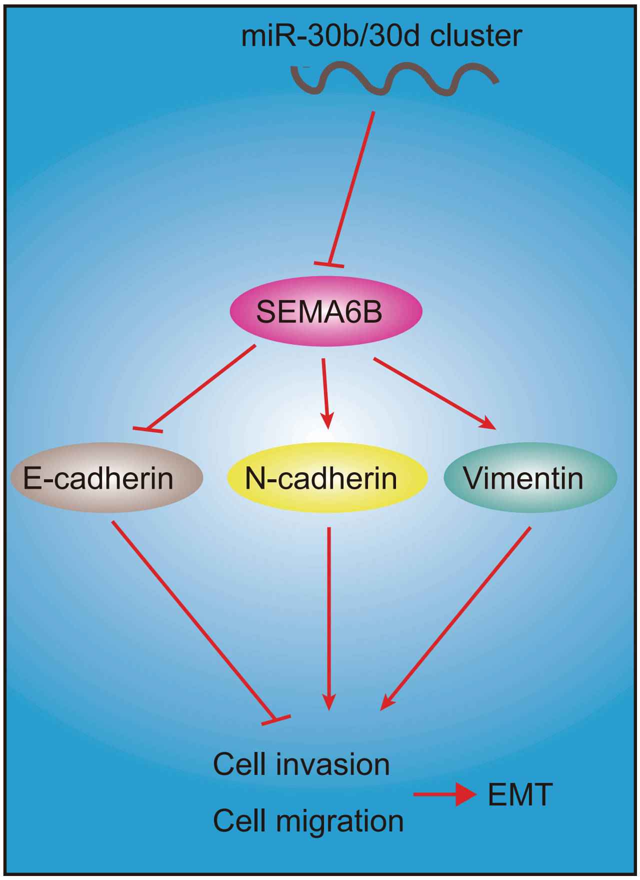

Figure 7. A diagram showing the mechanism by which miR-30b/-30d exerts synergistic effects on GBC progression

Overexpression of miR-30b/-30d inhibits GBC cell migration, invasion, and EMT process *in vitro* as well as weakening tumorigenesis of GBC cells *in vivo* through target inhibition of SEMA6B.

SEMA6B (Figure 7). The observations of our study provide evidence highlighting the conserved position markers as a promising preventive miRNA-based therapeutic strategy against GBC. Nevertheless, certain limitations were faced during the study, and considering the physiological and pathophysiological differences when relating the results of animal models to human clinical setting requires further study. Further human investigations are expected to determine the clinical significance of the reported axis.

MATERIALS AND METHODS

Ethics statement

All subjects enrolled into the study signed informed consent documentation. The study protocol was performed with the approval of the Ethics Committee of the Linyi People's Hospital. All animal experiments were conducted with the approval from the Institutional Animal Care and Use Committee of the Linyi People's Hospital. Extensive efforts were made to ensure minimal animal suffering as well as the number of animals used in the study.

Tissue specimen collection

Cancerous tissues were collected from 30 cases of GBC and gallbladder tissues from 18 cases with chronic cholecystitis. All patients were admitted to the Linyi People's Hospital between January 2018 and January 2019. GBC diagnosis was confirmed by means of histopathological evaluations, with none of the patents having received radiotherapy and chemotherapy prior to tissue specimen collection.

Isolation of HGBECs and culture of human GBC cell lines

HGBECs were established from donation, after brain death, of gallbladder based on a previously described method[31,32] and subsequently grown as monolayers. The epithelial nature of these cells was confirmed via immunohistochemical staining for CK19 (1:200, ab52625, Abcam, Cambridge, UK). Human GBC cell lines (purchased from Shanghai Institute of Nutrition and Health, Chinese Academy of Sciences), GBC-SD, Mz-ChA-1, NOZ, and SGC-996 were grown in the Roswell Park Memorial Institute (RPMI) 1640/fetal bovine serum (FBS) medium (HyClone, Logan, UT, USA) at 37°C, with exposure to an atmosphere of 5% CO_2.

Dual-luciferase reporter gene assay

The 3′ UTRs of SEMA6B wild-type (SEMA6B-WT) and SEMA6B mutant in seed sequence (SEMA6B-MUT) were inserted into the 3′ UTR of the luciferase reporter gene to construct recombinant vectors. The desired luciferase reporter plasmids, SEMA6B-WT or SEMA6B-MUT, were subsequently co-transfected with miR-30b mimic and miR-30d mimic into HEK293T cells. The luminescence of firefly luciferase was determined using a dual-luciferase reporter assay system kit (E1910, Promega, Madison, WI, USA) in accordance with the instructions provided by manufacturer, relative to that of Renilla luciferase.

RNA interference and plasmid delivery

The miR-30b mimic, miR-30d mimic, and miR-30b/-30d mimic (Shanghai GenePharma, Shanghai, China) were introduced into the GBC cells in an attempt to elevate the expression of miR-30b, miR-30d, and miR-30b/-30d using Lipofectamine 2000 reagents (11668-019, Invitrogen, Carlsbad, CA, USA) based on standard protocols, with the miRNA-mimic negative control (NC) regarded as the NC. A siRNA targeting SEMA6B and an expression vector containing the SEMA6B gene (both purchased from Shanghai GenePharma, Shanghai, P.R. China) were delivered into GBC cells to specifically blunt and overexpress SEMA6B, respectively, using Lipofectamine 2000 reagents (Invitrogen). A scramble siRNA or empty vector was used as the NC. Cell transfection was performed during a period of 24–48 h.

qRT-PCR

Total RNA was extracted from cells and tissues using the TRIzol reagent (Life Technologies, Gaithersburg, MD, USA). With regard to miRNA, complementary DNA (cDNA) was generated using a kit (#D1801) following the instructions provided by the manufacturer (HaiGene, Harbin, P.R. China). With regard to mRNA, the synthesis

Table 1. Primer sequences used for qRT-PCR

Target	Primer sequence (5′ → 3′)
SEMA6B	F: CTCTTTGTGTGCGGTTCCAA
	R: GAGCATCCCGTCAGAGAAGA
miR-30b	F: CGCGCTGTAAACATCCTACAC
miR-30d	F: TGTAAACATCCCCGACTGGAAG
Universal	R: GTGCAGGGTCCGAGGT
U6	F:GCTTCGGCAGCACATATACTAAAAT
	R: CGCTTCACGAATTTGCGTGTCAT
GAPDH	F: CCACATCGCTCAGACACCAT
	R: GCGCCCAATACGACCAAAT

of cDNA was performed using a kit (#K1622) based on the instructions provided by the manufacturer (Yaanda, Beijing, P.R. China). qRT-PCR was carried out using a qPCR instrument (ViiA 7, DaAn Gene, Guangzhou, P.R. China), with each reaction run in triplicate. The expression of target miRNA was normalized to the expression of U6, and the expression of target mRNA was normalized to the expression of glyceraldehyde-3-phosphate dehydrogenase (GAPDH). The results were calculated using the $2^{-\Delta\Delta Ct}$ method. The primers used in this assay are depicted in Table 1.

Immunoblot analysis

Protein extraction was performed using a protease inhibitor-contained radioimmunoprecipitation assay (RIPA) lysis buffer (Beyotime Biotechnology, Shanghai, P.R. China). After sodium dodecyl sulfate-polyacrylamide gel electrophoresis (SDS-PAGE) analysis, the protein was transferred onto polyvinylidene fluoride (PVDF) membranes and probed with the following primary antibodies (Abcam, UK): anti-SEMA6B antibody (1:1,000, ab180215), anti-N-cadherin antibody (1:1,000, ab18203), anti-E-cadherin antibody (1:500, ab15148), anti-vimentin antibody (1:1,000, ab137321), and anti-GAPDH antibody (1:2,500, ab9485) at 4°C overnight. The next day, the membranes were re-probed with horseradish peroxidase-coupled goat anti-rabbit immunoglobulin G (IgG) at room temperature for 1 h, after which immunoblots were then exposed using enhanced chemiluminescence detection reagents (EMD Millipore, Billerica, MA, USA). The gray values of the target protein bands were quantified using ImageJ software, with GAPDH used for normalization.

Immunofluorescence

Following paraformaldehyde fixation (4 h) and Triton X-100 incubation (15 min), the GBC cells were permitted to react with the aforementioned primary antibodies against E-cadherin, N-cadherin, and vimentin for immunoblot analysis. The results were subsequently visualized under a fluorescence microscope (Nikon, Tokyo, Japan).

Scratch test

Cells maintained with serum-free medium were seeded onto a six-well plate at a density of 5 × 10^5 cells/well. A thin scratch (10 μL) was created along the center of each well using a sterile pipette tip (the width of each scratch was the same). In order to evaluate wound closure, six fields were selected, and the cells were imaged at 0 hour and 24 h after incubation with serum-free medium. The cells in the wound area were counted and analyzed using counting software.

Transwell invasion assay

GBC cells were adjusted to a density of 5 × 10^5 cells/mL using serum-free RPMI 1640 medium with 100 μL of cell suspension added to the upper chambers coated with 30 μL Matrigel (YB356234, Shanghai Yubo Biological Technology, Shanghai, P.R. China) that had been diluted with serum-free Dulbecco's modified Eagle's medium (DMEM). After a 24-h incubation at 37°C, the cells that were transferred to the lower chamber containing 20% FBS-supplemented DMEM were subjected to 0.1% crystal violet staining and counted in six random fields per well using an inverted microscope (Olympus, Tokyo, Japan).

Tumorigenicity assay of human hepatocellular carcinoma (HCC) cells in nude mice

Specific pathogen-free (SPF) nude mice (6–8 weeks of age, n = 15, Hunan SJA Laboratory Animal, Hunan, China) were subcutaneously injected with human GBC cells treated with miR-30b/-30d mimic alone or in combination with expression vector containing the SEMA6B gene at a dose of 5 × 10^5 cells/mL. Tumor growth was examined on a weekly basis during a 6-week period. All mice were euthanized by means of cervical dislocation. Fresh tumor tissues were fixed and paraffin-embedded.

Immunohistochemistry

Paraffin-embedded cancerous and non-cancerous gallbladder tissues were cut into 5-μm-thick sections. The sections were subsequently constructed onto slides and immunostained for E-cadherin, N-cadherin, and vimentin using primary antibodies in line with those used for immunoblot analysis Visualization was performed using diaminobenzidine (DAB) (DA1010, Solarbio Science & Technology, Beijing, P.R. China). Five fields of view at ×200 magnification were randomly captured for each replicate using an inverted microscope (Nikon, Tokyo, Japan). The degree of staining was scored as 0 (0% of cytoplasmically stained cells); 1 (1%–25% of cytoplasmically stained cells); 2 (26%–50% of cytoplasmically stained cells); 3 (51%–75% of cytoplasmically stained cells); and 4 (76%–100% of cytoplasmically stained cells).

Statistical analysis

All data (mean ± standard deviation) were recorded based on the mean of three independent experiments (each in triplicate). An independent sample t test, a one-way analysis of variance (ANOVA) with Tukey's test, and repeated-measures ANOVA with a Bonferroni test were performed when appropriate for statistical comparisons. All statistical analyses were performed using SPSS 19.0 software (IBM, Armonk, NY, USA), with a two-tailed value of $p < 0.05$ considered to be indicative of statistical significance.

Data availability

The datasets generated and/or analyzed during the current study are available from the corresponding author upon reasonable request.

SUPPLEMENTAL INFORMATION

Supplemental Information can be found online at https://doi.org/10.1016/j.omtm.2020.11.019.

ACKNOWLEDGMENTS

We give our sincere gratitude to the reviewers for their valuable suggestions.

DECLARATION OF INTERESTS

The authors declare no competing interests.

REFERENCES

1. Zaidi, M.Y., and Maithel, S.K. (2018). Updates on gallbladder cancer management. Curr. Oncol. Rep. *20*, 21.
2. Bray, F., Ferlay, J., Soerjomataram, I., Siegel, R.L., Torre, L.A., and Jemal, A. (2018). Global cancer statistics 2018: GLOBOCAN estimates of incidence and mortality worldwide for 36 cancers in 185 countries. CA Cancer J. Clin. *68*, 394–424.
3. Sharma, A., Sharma, K.L., Gupta, A., Yadav, A., and Kumar, A. (2017). Gallbladder cancer epidemiology, pathogenesis and molecular genetics: recent update. World J. Gastroenterol. *23*, 3978–3998.
4. Li, Z., Yu, X., Shen, J., Law, P.T., Chan, M.T., and Wu, W.K. (2015). MicroRNA expression and its implications for diagnosis and therapy of gallbladder cancer. Oncotarget *6*, 13914–13921.
5. Chandra, V., Kim, J.J., Mittal, B., and Rai, R. (2016). MicroRNA aberrations: an emerging field for gallbladder cancer management. World J. Gastroenterol. *22*, 1787–1799.
6. Biswas, S. (2018). MicroRNAs as therapeutic agents: the future of the battle against cancer. Curr. Top. Med. Chem. *18*, 2544–2554.
7. Yang, L., Huang, S., Ma, H., Wu, X., and Feng, F. (2017). MicroRNA-125b predicts clinical outcome and suppressed tumor proliferation and migration in human gallbladder cancer. Tumour Biol. *39*, 1010428317692249.
8. Zhang, M., Gong, W., Zuo, B., Chu, B., Tang, Z., Zhang, Y., Yang, Y., Zhou, D., Weng, M., Qin, Y., et al. (2016). The microRNA miR-33a suppresses IL-6-induced tumor progression by binding Twist in gallbladder cancer. Oncotarget *7*, 78640–78652.
9. Mittal, V. (2018). Epithelial mesenchymal transition in tumor metastasis. Annu. Rev. Pathol. *13*, 395–412.
10. Liu, H., Zhang, N., and Tian, D. (2014). miR-30b is involved in methylglyoxal-induced epithelial-mesenchymal transition of peritoneal mesothelial cells in rats. Cell. Mol. Biol. Lett. *19*, 315–329.
11. Ye, Z., Zhao, L., Li, J., Chen, W., and Li, X. (2015). miR-30d blocked transforming growth factor β1-induced epithelial-mesenchymal transition by targeting snail in ovarian cancer cells. Int. J. Gynecol. Cancer *25*, 1574–1581.
12. Gaziel-Sovran, A., Segura, M.F., Di Micco, R., Collins, M.K., Hanniford, D., Vega-Saenz de Miera, E., Rakus, J.F., Dankert, J.F., Shang, S., Kerbel, R.S., et al. (2011). miR-30b/30d regulation of GalNAc transferases enhances invasion and immunosuppression during metastasis. Cancer Cell *20*, 104–118.
13. D'Apice, L., Costa, V., Valente, C., Trovato, M., Pagani, A., Manera, S., Regolo, L., Zambelli, A., Ciccodicola, A., and De Berardinis, P. (2013). Analysis of *SEMA6B* gene expression in breast cancer: identification of a new isoform. Biochim. Biophys. Acta *1830*, 4543–4553.
14. Kigel, B., Rabinowicz, N., Varshavsky, A., Kessler, O., and Neufeld, G. (2011). Plexin-A4 promotes tumor progression and tumor angiogenesis by enhancement of VEGF and bFGF signaling. Blood *118*, 4285–4296.
15. Letelier, P., García, P., Leal, P., Álvarez, H., Ili, C., López, J., Castillo, J., Brebi, P., and Roa, J.C. (2014). miR-1 and miR-145 act as tumor suppressor microRNAs in gallbladder cancer. Int. J. Clin. Exp. Pathol. *7*, 1849–1867.
16. Hua, C.B., Song, S.B., Ma, H.L., and Li, X.Z. (2019). miR-1-5p is down-regulated in gallbladder carcinoma and suppresses cell proliferation, migration and invasion by targeting Notch2. Pathol. Res. Pract. *215*, 200–208.
17. Zhou, N., Cheng, W., Peng, C., Liu, Y., and Jiang, B. (2017). Decreased expression of hsa-miR-372 predicts poor prognosis in patients with gallbladder cancer by affecting chloride intracellular channel 1. Mol. Med. Rep. *16*, 7848–7854.
18. Qin, X., Yu, S., Zhou, L., Shi, M., Hu, Y., Xu, X., Shen, B., Liu, S., Yan, D., and Feng, J. (2017). Cisplatin-resistant lung cancer cell-derived exosomes increase cisplatin resistance of recipient cells in exosomal miR-100-5p-dependent manner. Int. J. Nanomedicine *12*, 3721–3733.
19. Qi, Z., Zhang, B., Zhang, J., Hu, Q., Xu, F., Chen, B., and Zhu, C. (2018). MicroRNA-30b inhibits non-small cell lung cancer cell growth by targeting the epidermal growth factor receptor. Neoplasma *65*, 192–200.
20. Liao, W.T., Ye, Y.P., Zhang, N.J., Li, T.T., Wang, S.Y., Cui, Y.M., Qi, L., Wu, P., Jiao, H.L., Xie, Y.J., et al. (2014). MicroRNA-30b functions as a tumour suppressor in human colorectal cancer by targeting *KRAS*, *PIK3CD* and *BCL2*. J. Pathol. *232*, 415–427.
21. He, Y., Chen, X., Yu, Y., Li, J., Hu, Q., Xue, C., Chen, J., Shen, S., Luo, Y., Ren, F., et al. (2018). LDHA is a direct target of miR-30d-5p and contributes to aggressive progression of gallbladder carcinoma. Mol. Carcinog. *57*, 772–783.
22. Xiong, Y., Wang, Y., Wang, L., Huang, Y., Xu, Y., Xu, L., Guo, Y., Lu, J., Li, X., Zhu, M., and Qian, H. (2018). MicroRNA-30b targets Snail to impede epithelial-mesenchymal transition in pancreatic cancer stem cells. J. Cancer *9*, 2147–2159.
23. Wang, N., Xiang, X., Chen, K., Liu, P., and Zhu, A. (2018). Targeting of NT5E by miR-30b and miR-340 attenuates proliferation, invasion and migration of gallbladder carcinoma. Biochimie *146*, 56–67.
24. Xie, R., Wu, S.N., Gao, C.C., Yang, X.Z., Wang, H.G., Zhang, J.L., Yan, W., and Ma, T.H. (2017). MicroRNA-30d inhibits the migration and invasion of human esophageal squamous cell carcinoma cells via the post-transcriptional regulation of enhancer of zeste homolog 2. Oncol. Rep. *37*, 1682–1690.
25. Chen, D., Guo, W., Qiu, Z., Wang, Q., Li, Y., Liang, L., Liu, L., Huang, S., Zhao, Y., and He, X. (2015). MicroRNA-30d-5p inhibits tumour cell proliferation and motility by directly targeting CCNE2 in non-small cell lung cancer. Cancer Lett. *362*, 208–217.
26. Feng, F., Wu, J., Gao, Z., Yu, S., and Cui, Y. (2017). Screening the key microRNAs and transcription factors in prostate cancer based on microRNA functional synergistic relationships. Medicine (Baltimore) *96*, e5679.
27. Tran, N., Abhyankar, V., Nguyen, K., Weidanz, J., and Gao, J. (2018). MicroRNA dysregulational synergistic network: discovering microRNA dysregulatory modules across subtypes in non-small cell lung cancers. BMC Bioinformatics *19* (*Suppl 20*), 504.
28. Zhao, Y., Cui, X., Zhu, W., Chen, X., Shen, C., Liu, Z., Yang, G., Liu, Y., and Zhao, S. (2017). Synergistic regulatory effects of microRNAs on brain glioma cells. Mol. Med. Rep. *16*, 1409–1416.
29. Ge, C., Li, Q., Wang, L., and Xu, X. (2013). The role of axon guidance factor semaphorin 6B in the invasion and metastasis of gastric cancer. J. Int. Med. Res. *41*, 284–292.
30. Wang, N., Xia, S., Chen, K., Xiang, X., and Zhu, A. (2015). Genetic alteration regulated by microRNAs in biliary tract cancers. Crit. Rev. Oncol. Hematol. *96*, 262–273.
31. Galivo, F.H., Dorrell, C., Grompe, M.T., Zhong, Y., Streeter, P.R., and Grompe, M. (2015). Novel surface markers directed against adult human gallbladder. Stem Cell Res. (Amst.) *15*, 172–181.
32. Manohar, R., Li, Y., Fohrer, H., Guzik, L., Stolz, D.B., Chandran, U.R., LaFramboise, W.A., and Lagasse, E. (2015). Identification of a candidate stem cell in human gallbladder. Stem Cell Res. (Amst.) *14*, 258–269.

OMTM, Volume *20*

Supplemental information

The microRNA cluster miR-30b/-30d prevents tumor cell switch from an epithelial to a mesenchymal-like phenotype in GBC

Kang Cui and Xinyan Bian

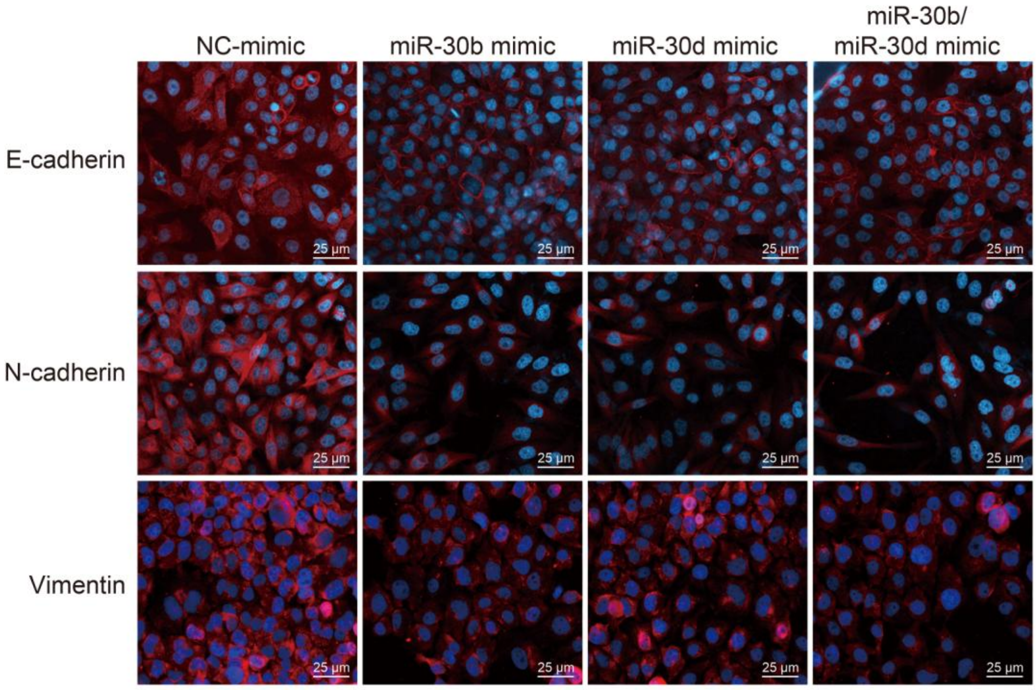

Supplementary Figure 1 Representative view of GBC-SD cells probed with antibodies against E-cadherin, N-cadherin, and vimentin and visualized by immunofluorescence (×400).
